# From training to practice: a report of professional capacity development in Health Research in West Africa

**DOI:** 10.1186/s12909-021-02696-7

**Published:** 2021-05-05

**Authors:** Issiaka Sombié, Sophie Fatoumata Bamouni, Donmozoun Télesphore Somé, Ermel Johnson, Jude Aidam

**Affiliations:** 1 West African Health Organisation, 175, Avenue Ouezzin Coulibaly, Bobo Dioulasso 01, 01 BP 153 Burkina Faso; 2 Institut Supérieur des Sciences de la Santé, Université Nazi Boni, Bobo-Dioulasso, Burkina Faso; 3 Société d’Etudes et de Recherche en Santé Publique (SERSAP), Ouagadougou 06, 06 BP 9150 Burkina Faso

**Keywords:** Capacity building, Research, Evaluation, West Africa

## Abstract

**Background:**

Between 2008 and 2013, the West African Health Organisation (WAHO) conducted a series of post-graduate capacity building in research methodology in West Africa. This work evaluated the contribution of these trainings in terms of knowledge acquisition and influence of research and policy practice. Cooke’s conceptual framework for assessing research capacity building was used with three data sources to construct the indicators (training reports, research project implementation reports and WAHO research programme evaluation report).

**Results:**

There was an improvement in the knowledge of the 84 participants between the pre- and post-test. At the end of the training, the learners developed 19 protocols, 14 of which were finalised, financed and implemented, reflecting the learners’ confidence to engage in research at the end of the training. The implementation of the protocols was conducted with the partnership and collaboration between the agents of the control programmes and the research centres. Some research results have been disseminated and a small portion used to strengthen the programmes.

**Conclusion:**

This evaluation showed that the training was linked to practice with little publication and use of the results to improve the programmes. This regional capacity building programme should be maintained and strengthened by adding modules in data analysis, scientific communication and knowledge transfer.

**Supplementary Information:**

The online version contains supplementary material available at 10.1186/s12909-021-02696-7.

## Background

High quality research is necessary for a better understanding of problems, its diagnosis and the development of an appropriate strategy for its resolution [1]. Moreover, for this research to be conducted, the skills needed are not often taught in training curricula and are acquired alongside experienced people. Thus, to enable health workers already in the field to acquire research skills, several non-academic training approaches have been applied in Africa with convincing results in terms of improving the capacities of actors, developing and implementing projects, and disseminating and using the results to improve programmes. However, it appears that most of the trainings were conducted outside West Africa [2–4]. One of the advantages of these non-academic training courses is that they allow for the training of practitioners and programme managers in a short time and help them apply this research to improve programme implementation [5]. However, to promote the success of these programmes, it has been suggested that research training be integrated into programme planning activities, accompanied by a supportive research environment (providing time for the programme staff to conduct research, collaboration with research structures, availability of financial incentives, funds to finance research projects, encouragement of dissemination and use of results) and an approach to promote staff retention [6].

In a systematic review of approaches and impact of non-academic research capacity strengthening training models in sub-Saharan Africa [4], only one training course was conducted in West Africa and it was more about training in ethics than in knowledge acquisition to develop protocols, apply them and disseminate the results to influence policy and practice. Also, this review noted a weak use of evaluation frameworks and few evaluation of dissemination of results. Finally, this review reported common challenges identified during these training including lack of mentorship and institutional support; insufficient time for research activities and drop out; lack of sufficient budget for research activities; poor research infrastructure; and difficulty in publishing in international journals. Thus, a good evaluation of these training programmes including knowledge and kills acquisition, research practice in the ground and dissemination of results with a knowledge transfer mechanism to influence policy and practice can help to better promote these research training programmes to improve health systems.

West Africa is recognised as the African region where health research is poorly conducted [7–11]. To help improve the situation, the West African Health Organisation (WAHO) has incorporated a programme for the development of research for health in its second strategic plan, 2009–2013 [12–14]. Within this research development programme, a capacity building strategy in health systems research methodology was implemented through the organisation of training workshops for health system actors. These workshops occurred from 2008 to 2013 in different countries of the sub-region and were aimed at strengthening the capacities of researchers and health workers in health systems research and giving them with opportunities to apply their knowledge through the development and implementation of research protocols and the dissemination of results. We sought to evaluate this training programme in terms of the acquisition of research knowledge and skills, the application of this knowledge in the field in terms of protocol writing, the conduct of research, the dissemination of results, the application of results in the field and costs. Specifically, we aimed to (i) assess the training cost and the acquisition of knowledge and skills by the trainees (ii) analyse the practice of research by the trainees after the training and (iii) study the process of dissemination of the research results and their use to influence programme implementation. These results can help in advocacy to improve the post academic training in research area.

This article is a report of the evaluation of this programme in terms of the acquisition of research knowledge and capacity, the application of this knowledge in the field in terms of protocol writing, the conduct of research, the dissemination of results, the application of results in the field and costs in the countries of the Economic Community of West African States (ECOWAS) and lessons learnt to help other actors.

## Methods

### Scope of the study

A review by Mugabo noted that several approaches have been used for the evaluation of post-graduate research capacity building programmes in Africa [4]. Our work used Cooke J’s conceptual framework [15], which allows us to see the contribution of training to learners’ knowledge, the practice of post-training research, collaboration and support to researchers, and the dissemination and use of results. This conceptual framework defines six principles. The first is the development of individual capacity and confidence through training and the creation of opportunities to apply this capacity. The second principle is the need to ensure that this training is linked to practice. The third principle is the development of partnerships and collaborations. The fourth principle is the need to ensure adequate dissemination of results. The fifth principle is to ensure continuity and sustainability and the sixth principle is the need for adequate infrastructure. We have adopted this conceptual framework because it covers all areas from the acquisition and application of knowledge and the contribution of this application of knowledge in terms of public health. Thus, the following six areas were considered when evaluating a research training programme: capacity development and individual confidence, practice-related training, strengthening partnerships and collaborations, adequate dissemination of results, continuity and sustainability, and the existence of adequate infrastructures. We used this conceptual framework to evaluate our post-graduate research capacity building programme.

We defined four indicators to assess capacity improvement and individual confidence (the number of participants with less than 50% correct answers, the number of participants with 50% or more correct answers in the pre- and post-test, the average knowledge of participants in the pre- and post-test, and the protocol development after training). Practice-related training was assessed by the involvement of trainees in research after training (number of people involved in research after training). Strengthening of partnership and collaboration was assessed by the existence of partnership and collaboration after training. Adequate dissemination was assessed by four indicators (production of scientific articles, conducting scientific communication at conferences, organization of workshops to disseminate results and experiences in using the results). Continuity and sustainability were assessed through access to funding after training to conduct research. Finally, the existence of adequate infrastructure was assessed by the existence of institutional support to conduct research after training.

### Data collection

Several sources of information were used to construct the indicators. The first source was the reports of the training workshops. The second source of data used was the implementation reports of the funded research protocols. The third source was the results of an evaluation of the WAHO research programme carried out by an independent consultant. The consultant, after reviewing all project documents and reports, interviewed some of the beneficiaries and other stakeholders of the research programme, including some of the people who had been trained in research methodology. The method of data collection was described in the previous publication [12]. The information from these three data sources was put together to construct the indicators of the six areas of the Cooke framework for each training conducted. Two persons extracted the information from these documents to construct the indicators.

The data for the three indicators to assess the improvement of the knowledge of the trained people were taken from the reports of the training workshops where the information on the pre-test and post-test evaluations was recorded. These data made it possible to determine the number of people with < 50% correct answers, the number of people with ≥50% correct answers and the average of correct answers (%) before and after the all training. The number of protocols received from the trainings and the protocols funded was obtained from the final report of the programme. The number of people involved in research after training, existence of partnership and collaboration after training, existence of institutional support to conduct research after training production of scientific articles, conducting scientific communication at conferences, organization of workshops to disseminate results and the use of the results were extracts on the project implementation report and the programme evaluation report.

## Results

### The description of the research methodology training programme

Table 1 presents the different training courses conducted in terms of location, trainers, participants, basic documents used for the training and cost of the training. It was noted that the trainings all lasted for 5 days. It was an adult training. Three type of tools were used: i) the WHO/IDRC research was used for trainings held in 2008 (Bobo-Dioulasso) and in 2012 (Lomé); ii) a document developed by a Gambian non-governmental organization (NGO) used in 2009 (The Gambia) and in 2012 (Liberia) and finally iii) a training document developed in Guinea Bissau. These documents were used according to the trainers who were from three groups. These trainers were senior researchers with experience in research and in training young researchers either at the level of the United Nations Joint Programme for Research on Tropical Diseases or at the country level. However, none of them taught health systems research in a university. Three groups of trainers were noted and each group used a different training tool.

**Table 1:** List of Training Courses organised in Health Systems Research by WAHO from 2008 to 2012 in ECOWAS countries

**Title**	Training of researchers and managers of national programmes in health systems research methodology	Training of researchers and programme managers in Liberia and Sierra Leone in health systems research methodology	Training of health actors in Guinea Bissau in health systems research methodology	Training of health actors in Guinea Bissau in health systems research methodology	Training of Liberian researchers and programme managers in health systems research methodology
**Description**	Five days of training in writing health systems research protocols	Five days of training in writing health systems research protocols	Five days of training in writing health systems research protocols	Five days of training in writing health systems research protocols	Five days of training in writing health systems research protocols
**Training period**	November 2008, 3 to 7	November 2009, 2 to 6	March 2012, 22 to 26	June 2012, 11 to 15	December 2012
**Place of training**	Bobo, WAHO headquarters	Banjul, Gambia, CIAM headquarters	Bissau, INASA Headquarter	Lomé, Epidemiology Directorate	Gbanrga, Liberia
**Training language**	English, French	English	Portuguese	French	English
**Number of trainees**	30	6	16	20	12
**Trainees profile**	Programme managers and researchers from the 14 ECOWAS countries	Liberia and Sierra Leone Researchers and programme Officers	Health workers (doctors, nurses, laboratory technicians, sociologist)	Health workers (doctors, biologists, nurses)	Researchers and programme officers Liberia
**Training materials**	WHO/IDRC Document	Social and economic research in tropical diseases resource paper no.3. (TDR/SER/RP/94.2)	INASA Document	WHO/IDRC Document	Social and economic research in tropical diseases resource paper no.3. (TDR/SER/RP/94.2)
**Trainers**	Research Director Senior Researcher	Two researchers working in a research institution and a financial manager	Six national researchers	Research Director Senior Researcher	Two researchers working in a research institution
**Training cost**	76,873 US $	24,153 US $	29,057 US$	33,142 US$	30,630 US$

The content of the programme revolved around the identification of research problems and questions, the definition of objectives, the development of research methodology, the development of a research timeline, the development of a research budget, selection and analysis of a health problem, ethical issues, and bibliographical references.

The health authorities of the country concerned designated the beneficiaries of these training programmes. They were researchers working in research institutions, programme managers at the level of the ministries of health and health officials at the level of the regions and health districts. The profiles of those trained included doctors, biologists, sociologists, laboratory technicians and nurses.

The training involved 30 persons for the first group in 2008 in Bobo-Dioulasso (Burkina Faso), 6 persons for the second group in 2009 in Banjul (The Gambia), 16 persons for the third group in 2010 in Guinea Bissau, 20 persons for the fourth group in 2012 in Lomé (Togo) and 12 persons in Liberia in 2012 for 84 persons.

The cost of the training varied from one workshop to another. It ranged from US$ 76,873 for the 2008 workshop in Bobo-Dioulasso to US$ 24,153 in Gambia, 33,142 in Lomé, 30,629 in Monrovia and 29,057 in Bissau with a total cost of US$ 226,996. The average cost of training per person trained was US$ 2562 in Bobo-Dioulasso, US$ 4025 in Banjul, and US$ 1816 in Bissau, US$ 1657 in Lomé and US$ 2552 in Monrovia, with an average cost per person trained of US$ 2702.3. The costs of the trainings included motivation costs, per diem of the trainees and trainers, trainers’ emoluments, travel costs of the trainees and trainers, room rental and training materials.

### Contribution of the training courses

Table 2 presents the training achievements according to the six areas of the Cooke J conceptual framework. It can be noted that the information was obtained for four of the five training courses conducted. The contribution of the training was therefore analysed on the basis of these four training courses. The training in Bissau was the one where information was not available.

**Table 2:** The evaluation details of the 5 training courses according to the six principles of the Cooke framework

Principles	Indicators	Bobo-Dioulasso 2008		Banjul 2009		Bissau, 2010		Lome, 2012		Gbanrga, 2012	
		Pre test	Post test	Pre test	Post test	Pre test	Post test	Pre test	Post test	Pre test	Post test
Capacity and confidence building	Number of people with < 50% correct answers	15	8	4	2			11	1	3	1
	Number of people with ≥50% correct answers	12	19	2	4			8	18	9	10
	Average of correct answers (%)	43	56	45	52			43,5	61,5	65,8	88,6
	Research companies after training	13 protocols developed and 11 implemented		Two protocols developed and one implemented				Four protocols developed and two implemented			
Practice-Related Research	Involvement of staff in research (number or proportion of people involved in research after training)	22 trained agents undertook research after training		2 trained agents undertook the research				10 trained agents undertook research after training			
Enhanced Research through Partnership	Between the new and the experienced	Yes Collaboration between researchers and programme manager		Yes Collaboration between researchers and programme manager				Yes Collaboration between researchers and programme manager			
	Strengthening the interprofessional link	Yes		Yes				Yes			
Impact of dissemination	Articles published in journals	Two articles published									
	Conference Papers										
	Dissemination workshops	Two dissemination workshops									
	Results used	Three experiences in using the reported results									
Continuity and sustainability	Access to financing	13 funded protocols		1 funded protocols				2 funded protocols			
Research Infrastructure	Institutional support to undertake research	Yes		Yes				Yes			

### Development of participants’ knowledge and confidence

We note that the number of people with 50% or more correct answers to the pre-test was less than half of the participants, except in Monrovia where 8 of the 12 participants were scored. This number at the post-test in all countries was more than half of the participants and was 10/12 in Monrovia. Similarly, the average correct pre-test response was less than 50% at each training level except in Liberia where it was 65.11%. In the post-test, it was noted that the average of over 50% correct answers varied in all trainings and ranged from 52% in Banjul to 88% in Monrovia. Generally, these results reflect an improvement in participants’ knowledge after the training, which may reflect the acquisition of new knowledge and skills in the field of research. Thus, it can be concluded that the different training courses have contributed to an improvement in the knowledge and skills of the participants.

### Continuity and sustainability

During three training workshops (Bobo-Dioulasso, Banjul and Lomé), the development of research protocols was initiated. These protocols should be finalised and, depending on quality, funded. In total, during the 2008 workshop in Bobo-Dioulasso, out of 15 protocols initiated, 13 were finalised and deemed suitable for funding by the trainers. In 2008, in Banjul, out of two protocols initiated, one was finalised and funded. During the 2012 workshop in Lomé, out of 4 protocols initiated, 2 were finalised and financed. At the end of the 2010 workshop in Bissau and the 2012 workshop in Liberia, no research protocol was finalised. These results reflect the confidence of the learners to engage in research at the end of the training. Thus, 54 (64.3%) of the 84 trainees participated in research after their training.

### Practice-related training

Out of the 16 protocols funded, 14 have been fully implemented and reported to the West African Health Organisation. These were 11 out of 13 from the 2008 workshop in Bobo-Dioulasso, 1 out of 1 from the 2009 workshop in Banjul and 2 funded projects from Lomé in 2012. The reasons for non-implementation of the two protocols funded after the 2008 Bobo-Dioulasso workshop were the use of funds for another activity and the retirement of the principal investigator associated with financial management difficulties.

The themes of the research protocols were parent-child communication for HIV/AIDS prevention, the importance of tuberculosis in HIV-infected people, the efficacy of artemisinin-based antimalarial drugs, factors associated with high reporting of typhoid fever, and the prevalence of dengue fever, the additional workload through decentralization of the care of people infected with HIV, neonatal infections in maternity wards, dysfunction and acts of non-quality in emergency services, the impact of reforms on the care of the elderly, the contribution of the introduction of financial motivation of health personnel on the supply of services.

### Strengthening partnership and collaboration

The training held in 2008 in Bobo-Dioulasso helped create a partnership between programme managers and researchers. Indeed, each country was asked to send one manager and one researcher to the training. During the training, the protocol was initiated based on a problem identified at the programme level. The protocols developed were implemented jointly and contributed to strengthening the partnership and collaboration between researchers and programme managers. In addition, the studies funded after the Banjul and Lomé training were implemented with collaboration between research structures and programme staff.

### Adequate dissemination of research results

Following the completion of these various studies, fourteen implementation reports were submitted to WAHO. Two projects were the subject of a workshop for sharing results at the national level, two were the subject of publication of scientific articles and two approach of use of research results were reported. The first approach is the use of the results to develop a national programme for the control of sickle cell disease in Niger. The second approach of use is the improvement of the organization of emergency services at the level of university hospitals in Côte d’Ivoire.

### Strengthening research infrastructure

The teams that implemented the research indicated that they had benefited from institutional support to implement their research. This involved support in managing the funds received, obtaining the necessary authorization and integrating research activities into the structures’ programmes of activities.

### The appreciation of the training by the learners

At the end of the 2008 workshop, a questionnaire allowed the final evaluation of the course by the participants. Out of 27 answers, all the participants found the workshop useful because it allowed the reinforcement of skills, the putting into practice of knowledge and will give an impulse to the use of research results. All participants said that the course would be useful once they return home. While most respondents considered the number of facilitators appropriate, some participants, wished to have more facilitators who spoke mainly English because the participants were French, English and Portuguese speakers. The level of the course was deemed adequate by 26 participants and too basic by one participant. The quality of the teaching was judged excellent (9 respondents), very good (13 respondents) and acceptable (5 respondents). Contact and interaction between participants was most often rated as insufficient (5 respondents), satisfactory (17 respondents), and excellent (4 respondents). Contact and exchanges between participants and facilitators were rated satisfactory (15 respondents) and excellent (10 respondents). All participants considered the process of the research proposal development useful. Concerning the duration of the course in working days, nine participants felt that it should be 5 days, for nine others it should be 15 days and for seven it should be 7 days. Most participants deplored the lack of Internet access in the training room for conducting the literature review.

## Discussion

This evaluation shows that the post-graduate research capacity building programme promoted by WAHO has contributed to improving the knowledge of participants, to the application of knowledge acquired through research practice and to a low level of dissemination and use of results to improve the implementation of health programmes in West Africa, with training costs varying from one country to another.

It is recognized that one of the barriers to research capacity building is a low budget allocation [4]. By providing information on the cost of training, our work can help in the future planning of research training. Training costs included motivation costs, trainees and trainers’ perdiems, trainers’ emoluments, trainees’ and trainers’ travel expenses, room rental and training materials. Our results showed different costs from one training course to another. The highest costs were noted for training courses that required travel of trainees and/or trainers (Bobo-Dioulasso, Banjul, and Monrovia). However, one of the advantages of moving trainees is that it facilitates their commitment and concentration on the training site. Alternatively, the costs of our training courses were lower than the costs reported by Zachariah et al. [3] for whom a training of 12 people with three modules was about 75,000 euros (90,000 US$) or 6250 euros (about 7500 US$) per person trained. It should also be noted that Zachariah et al.’s team training was spread over a period of 10 to 12 months.

The review of post-graduate research training in Africa had shown evidence that these trainings had contributed to improving the knowledge and skills of the trainees [4]. Our results confirm this evidence with more than half of the participants in our training having knowledge scores of more than 50% correct answers.

Our results showed that practice was linked to training, as most of the protocols initiated during training were finalised, funded and implemented. Factors limiting the transition from training to research practice include lack of time for researchers after training, lack of funding for projects developed, and lack of supervisors and mentors [4, 16]. By involving programme managers and researchers in our training, and by setting up funds to finance research projects finalized after training, our approach has reduced these barriers, which has allowed most of the projects initiated during training to be finalized and implemented.

Concerning the implementation of research projects, among the projects resulting from the training sessions, 13 (68.4%) could be implemented, only the results of 2 were the subject of a national dissemination sharing workshop, the results of 2 were the subject of publication of scientific articles and the results of 2 were used for the implementation of the national programme of fight against sickle cell disease in Niger. Thus, if practice has been associated with research in our work as in the programme developed by Zachariah et al. [3], the number of projects leading to publications was low in our study. The elements of success identified by Zachariah et al. explains the difference noted between the two works. Indeed, in the work of Zachariah et al., the criteria for selecting participants were favorable to motivated participants and there were deadlines and milestones to be reached with the presence of experienced facilitation mentors. In addition, Zachariah et al.’s programme was spread over 10 to 12 months with two additional modules in data analysis and scientific writing. Therefore, for Mugabo et al. [4], a long period of programme implementation, in addition to technical and financial support to complete research projects, is likely to increase the number of protocols developed, research conducted and publications produced, as well as influence policy and practice. Thus, future WAHO training programmes should consider the need to integrate training modules in data analysis and scientific writing.

WAHO, to strengthen the research field in West Africa, organised this training programme for researchers in research methodology and provided financial support to some research projects resulting from these trainings. According to Nchinda in 2002 [17], the strengthening of research is based on two closely interrelated and interdependent principles: institutional support and the improvement of researchers’ capacities [6]. WAHO has therefore well integrated these two principles for the strengthening of research in West Africa, but the training provided was not complete because it did not provide skills in data analysis and scientific writing, skills crucial for the publication of results to influence policies and practices. It would be equally important nowadays to include a module on knowledge transfer given the weak skills of research stakeholders in West Africa [18].

Of course, the contribution of WAHO in the regional training space is commendable but this action falls short of expectations. The situation differs according to the poles, i.e., between the Francophone, Anglophone and Lusophone regions. As the results of the evaluation showed, few health workers have a research culture. This is perceptible among executives involved in the decision-making sphere and programme implementation players and clinicians. However, there is evidence that the training of health workers can have a considerable impact on the health system as a whole [19]. This means that training in research should be intensified; the research departments in the ministries of health of the various countries must implement such training to reach the maximum number of personnel. Priority should be given to decentralised actors in the health system such as district chief medical officers and regional health directors. According to Zwanikken et al. [19], trained middle-level health managers are better able to be leaders and conduct research activities, all of which can benefit their institutions and the entire health system. In addition, it has been shown in Rwanda that series of research training seminars have been opportunities for engaging clinicians and non-clinicians in research and have served as a starting point for advanced research training [20].

Learners appreciated the importance of the training initiated by WAHO. The same was true of the training described by Mahendradhata Y et al. [21]. This research also raises the need for systematization of training. In addition, various concerns were raised by participants in these trainings, including the number of days of training, the need for accompaniment, etc. [22]. Thomson et al. [22] highlights the important lessons to consider when initiating research capacity building trainings in middle and low-income countries. These lessons concern the determination of a favorable period, the integration of training in the work plan, the continuous accompaniment of a mentor, the rigor in the selection, the investment in the material necessary for the training and consideration of local institutions for the implementation of the trainings. During the trainings, undertaken, similar actions have been implemented; the important lesson from our perspective is accompaniment; this is all the more important because the process of acquiring and using new knowledge is unsystematic. This is what makes it important to consider local institutions.

It therefore remains for WAHO, as a regional institution, to consider the needs expressed and work to improve the level of health research capacities in different countries. The institution’s Regional Strategic Plan for Health Research 2016–2020 has made a provision for continuing to train health actors in research and to support them in applying this knowledge to produce and use the results in improving the provision of care to populations. This can only be done by developing, in partnership with the states, policies for training middle managers, which can be real levers for bridging the gap compared to other regions of the continent. However, none of this is possible unless the political will is present and funding is available. The costs of the various training courses are high, but strategies exist to make these courses more efficient, which will benefit more players.

Our study has limitations, particularly in terms of the completeness of the data. Indeed, having used secondary data, it was not always possible to obtain the necessary information for the indicators in the context of certain training courses. This weakness is known in studies using secondary data. Despite this weakness, our results provide information that can help improve training.

## Conclusion

Our work has shown that WAHO’s post-graduate training programme in health research has improved the knowledge, skills and practices of health workers. Dissemination and support for the use of results to influence policy will need to be improved. For example, future training should include modules on knowledge analysis and knowledge transfer, targeting health workers in programmes or services whose results can help bring about change.

## Supplementary Information

**Additional file 1.**

## Data Availability

The datasets generated and/or analyzed during the current study are available on demand basis from the corresponding.
