# Supplementary material for: From training to practice: a report of professional capacity development in Health Research in West Africa
Source: BMC Med Educ. 2021 May 5;21:259. doi: 10.1186/s12909-021-02696-7 (PMC8101165; doi:10.1186/s12909-021-02696-7)
Supplement: Supplementary file 1 — Additional file 1. [file 12909_2021_2696_MOESM1_ESM.doc]

**From Training to Practice: An Experience of Professional Capacity Development in Health Research in West Africa**

*Issiaka Sombié^1, 2^, Sophie Fatoumata Bamouni^2^, Donmozoun Télesphore Somé^3^, Ermel Johnson^1^, Jude Aidam^1^*

1. West African Health Organisation, BP 153 Bobo-Dioulasso, Burkina Faso

2. Institut Supérieur des Sciences de la Santé, Université Nazi Boni, Bobo-Dioulasso

3. Société d'Etudes et de Recherche en Santé Publique (SERSAP), 06 BP 9150 Ouagadougou 06 Burkina Faso

Corresponding author: Professor Issiaka Sombié, MD, PhD. West African Health Organisation (WAHO), 175, Avenue Ouezzin Coulibaly, 01 BP 153 Bobo Dioulasso 01, Burkina Faso

**waho Workshop ON METHODOLOGY of research in health**

**Pre-test/Post -test**

**Participant’s number: ..... /_/_/**

**Date: /_/_/ - /_/_/ - /_/_/_/_/**

*Read carefully and tick the appropriate answer (S) for each question*

*A wrong answer invalidates the appropriate answer (S) for the same question*

**1. List 4 main components of a research project**

-

-

-

-

**2. The formulation of a research topic must help:**

- To clearly define the nature of the problem the topic is all about
- to review the current development about the issue
- to predict in simple and precise terms, the expected results
- to identify the variables to be measured

**3. The selection criteria of a research topic are based in priority on:**

- Relevance
- Innovation
- Feasibility
- deontological and ethical Acceptability

**4. A variable is a characteristic of an object, a phenomenon or person that can take several values**

- True
- False

**5. the specific objectives that clearly identify what should be done to solve a problem, are mutually exclusive**

- True
- False

**6. The following types of study are descriptive studies that help conduct a particular situation analysis**

- Longitudinal
- Randomized
- Textbook Case
- Cross-cutting or cross-disciplinary

**7. The following indicators are numerical variables**

- Height
- Gender
- Age
- Nationality

**8. The formulation of the objectives helps**

- to define the essential scope of the study
- not to collect unnecessary information
- to describe the types of study to be carried out
- to have an overview of the tasks to be performed

**9. A research project must base on a literature review in order:**

- not to reinvent the wheel
- to argue the justification of the research project
- to make a good choice of methodology
- to establish a link between a factor and the issue of the study

**10. A good objective must have the following characteristics**

- Specific
- Measurable
- Achievable
- Realistic
- Time-Related

**11. Experimental studies help:**

- To prove causality
- To compare the effect of an intervention in the 2 groups
- To discover the factors underlying the problem
- To determine the causes or risk factors

**12. List 4 data collection techniques** -

-

-

-

**13. Observation is a data collection technique in which the following data collection tools are used:**

- Questionnaire
- Microscope
- DNA Amplifier (thermocyclor)
- Spectrophotometer

**14. Sampling consists in choosing part of a group or population to get a piece of information that can be generalized to the whole population.**

- True
- False

**15. A sample must help to reduce the costs and time of study implementation**

- True
- False

**16. List one non-probability and three probability sampling methods**

-

-

-

-

**17. In a very large population (eg: population of Nigeria) very dispersed and diversified** **where it is impossible to draw up the list of the people per area (village/town), the following sampling method is recommended:**

- Random sampling
- Systematic sampling
- Convenience sampling
- Cluster sampling with one or several degrees

**18. The sample size is generally based on the main purpose of the study**

- True
- False

**19. A good data collection plan allows:**

- To have a good overview of the tasks to be performed and the duration of the activities
- To organize the necessary human and material resources
- To minimize the errors and delays resulting from an insufficient planning
- To assess the sample size

**20. A Work plan is a calendar, table or diagram clearly summarizing the various elements of a project and the links between one and another**

- True
- False
